# The Art of Eating

**Published:** 1909-02-20

**Authors:** 


### The Art of Eating.

Yet another great volume upon dietetics!* Certainly civilised humanity has no excuse for not ordering its food and feeding according to approved scientific principles; yet this same humanity remains remarkably unmoved by the cataract of wisdom which has lately descended upon its head. We do not observe any inclination to determine menus by the calorie-value of their ingredients, nor any diffuse tendency to fly the onslaught of purins in general and uric acid in particular. The truth is, we fancy, that people in health find dietetic subtleties rather boring, and soon come to the conclusion that the business is overdone. They are perfectly prepared to admit the justice of the old motto, "Moderation in all things," but restrictions going beyond this modest limit are "fads," and as such to be laughed at. We may frankly say that we believe they are right, and that, in health, no one needs more advice upon diet than Bacon offered centuries ago. "There is," he says, "a wisdom in this (*i.e.* 'The Regimen of Health') beyond the rules of physic; a man's own observation, what he finds good of, and what he finds hurt of, is the best physic to preserve health.' But it is a safer conclusion to say, 'This agreeth not well with me, therefore I will not continue it,' than this 'I find no offence of this, therefore I may use it.' For strength of nature in youth passeth over many excesses which are owing a man till his age. Discern of the coming on of years, and think not to do the same things still; for age will not be defied." And again, "To be free-minded and cheerfully disposed at hours of meat and sleep and of exercise is one of the best precepts of long-lasting." Nothing could better this advice, and nothing more than this is required by the healthy individual. Indeed, one may go further and say that curiosity and attention to diet carried beyond this stage are good evidence of uneasiness as regards his health on the part of him who manifests these symptoms; and the fact that food fads among the apparently healthy have attained their present proportions is proof positive that a large number of us live in that vague borderland between health and illness where no definite ailment exists, but sense of well-being is deficient.

These somewhat derogatory observations notwithstanding, we are profoundly convinced of the value derived from the scientific investigation of dietetics. This study has exploded much fallacious tradition, and, if it has left a good many problems unsolved, has to a large degree replaced assumptions by facts. In no domain of dietetics has this influence been so great as in that which has to do with alcohol. Supporters of the use of alcohol have made a great parade of the fact that it is a food, and a good deal of special pleading has been based upon this fact. But we need no longer deceive ourselves on the matter. It is true that alcohol is a food in the strict sense, in that it is oxidised in the body, and is in some cases of great value on this account owing to the rapidity of its absorption. But it is thoroughly established that, considered as an ordinary article of diet, alcohol is a luxury pure and simple, whose good effect is psychical (in so far as it promotes good fellowship) and not alimentary, and whose evil influences lie not far below the surface.

The book before us is somewhat over-long. For example, Dr. Harry Campbell's article on the evolution of man's diet is entertaining, but not helpful, while the dietetic directions under the heading of several special diseases are profoundly platitudinous and not worth insertion. But on the whole the work is a complete and good book of reference. We would direct especial attention to Dr. Harry Campbell's article on alcohol, and the reasons which underlie the universal popularity of the drug. His speculations regarding the presence in the blood of substances which determine the emotional tendencies of individuals are extremely suggestive. He argues that in health the blood contains both stimulant and depressant agencies, the relative proportions of which dictate the sense of well-being or ill-being of the individual for the time; and that the popularity of alcohol depends upon its power of fulfilling, for the moment, the action of the hypothetical body which makes life joyous. Could the physiologists but find us this elixir, what an armament would be added to medicine!

* "A System of Diet and Dietetics." Edited by G. A. Sutherland. (London: Oxford Medical Publications. 1908. Pp. 893. 30s. net.)

